# A genotyping‐in‐thousands by sequencing panel to inform invasive deer management using noninvasive fecal and hair samples

**DOI:** 10.1002/ece3.8993

**Published:** 2022-06-10

**Authors:** Brock T. Burgess, Robyn L. Irvine, Michael A. Russello

**Affiliations:** ^1^ Department of Biology The University of British Columbia Kelowna Canada; ^2^ Ecosystem Conservation Team Protected Areas Establishment and Conservation Directorate Parks Canada Agency Gatineau Canada

**Keywords:** conservation genetics, GT‐seq, invasive species, molecular ecology, noninvasive genetic sampling, single nucleotide polymorphism

## Abstract

Studies in ecology, evolution, and conservation often rely on noninvasive samples, making it challenging to generate large amounts of high‐quality genetic data for many elusive and at‐risk species. We developed and optimized a Genotyping‐in‐Thousands by sequencing (GT‐seq) panel using noninvasive samples to inform the management of invasive Sitka black‐tailed deer (*Odocoileus hemionus sitkensis*) in Haida Gwaii (Canada). We validated our panel using paired high‐quality tissue and noninvasive fecal and hair samples to simultaneously distinguish individuals, identify sex, and reconstruct kinship among deer sampled across the archipelago, then provided a proof‐of‐concept application using field‐collected feces on SGang Gwaay, an island of high ecological and cultural value. Genotyping success across 244 loci was high (90.3%) and comparable to that of high‐quality tissue samples genotyped using restriction‐site associated DNA sequencing (92.4%), while genotyping discordance between paired high‐quality tissue and noninvasive samples was low (0.50%). The panel will be used to inform future invasive species operations in Haida Gwaii by providing individual and population information to inform management. More broadly, our GT‐seq workflow that includes quality control analyses for targeted SNP selection and a modified protocol may be of wider utility for other studies and systems where noninvasive genetic sampling is employed.

## Graphical abstract

Molecular ecology employs noninvasive sampling that can pose challenges for massively parallel DNA sequencing, with current methods limited in data quality/applicability. We demonstrate a GT‐seq workflow that can be applied to fecal and hair samples with high sample retention and genotyping accuracy, and provide a proof‐of‐concept application to invasive species management.

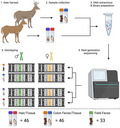

## 1 INTRODUCTION

Genotyping greater numbers of loci for studies in ecology, evolution, and conservation has important advantages, from gaining the ability to address more complex questions to reinforcing fundamental concepts with superior statistical power (reviewed in Hohenlohe et al., 2021). However, molecular studies involving elusive or at‐risk species often rely on noninvasive samples (e.g., feces, hair, or feathers) that can pose challenges for massively parallel DNA sequencing because of poor DNA quality, low DNA quantity, and exogenous DNA contamination (Andrews et al., 2018; Russello et al., 2015). Such impediments are now starting to be overcome, driven in part by multiplexed targeted amplicon sequencing (MTAS) approaches (Eriksson et al., 2020; Hayward et al., 2022; Natesh et al., 2019; Schmidt et al., 2020). MTAS can target hundreds of single nucleotide polymorphisms (SNP) within a single PCR to genotype thousands of individuals in parallel (Campbell et al., 2015), yet genotyping success and sample retention has varied widely when applying these approaches to noninvasive samples (Eriksson et al., 2020; Hayward et al., 2022; Natesh et al., 2019).

While low costs and fast turnaround times have been highlighted as major advantages of MTAS for applications in conservation and molecular ecology (Campbell et al., 2015; Natesh et al., 2019), a unique aspect of these approaches is their scalability, wherein investigators can strategically include targeted loci that allow SNP panels to simultaneously provide different types of relevant information (e.g., individual/species identification, sex, population assignment). Such designs have been demonstrated in several studies using traditional genetic samples (Bootsma et al., 2020; Chang et al., 2021; May et al., 2020; Sjodin et al., 2020), but have been limited for those employing noninvasive sampling, especially regarding the number of loci and/or samples genotyped as well as the form of starting material. For example, Eriksson et al. (2020) demonstrated high levels of genotyping success and sample retention using coyote feces, but only multiplexed a small number of loci (*n *= 26). In contrast, Hayward et al. (2022) successfully genotyped several hundred loci using polar bear feces, but experienced only ~60% sample retention even following initial sample screening using species‐specific qPCR. Importantly, such studies employing MTAS have been largely limited to fecal samples despite the fact that hair remains a valuable DNA source for many mammalian systems dating back to the first studies employing noninvasive genetic sampling (Andrews et al., 2018; Morin & Woodruff, 1992; Taberlet & Bouvet, 1992; Waits & Paetkau, 2005).

**FIGURE 1:**
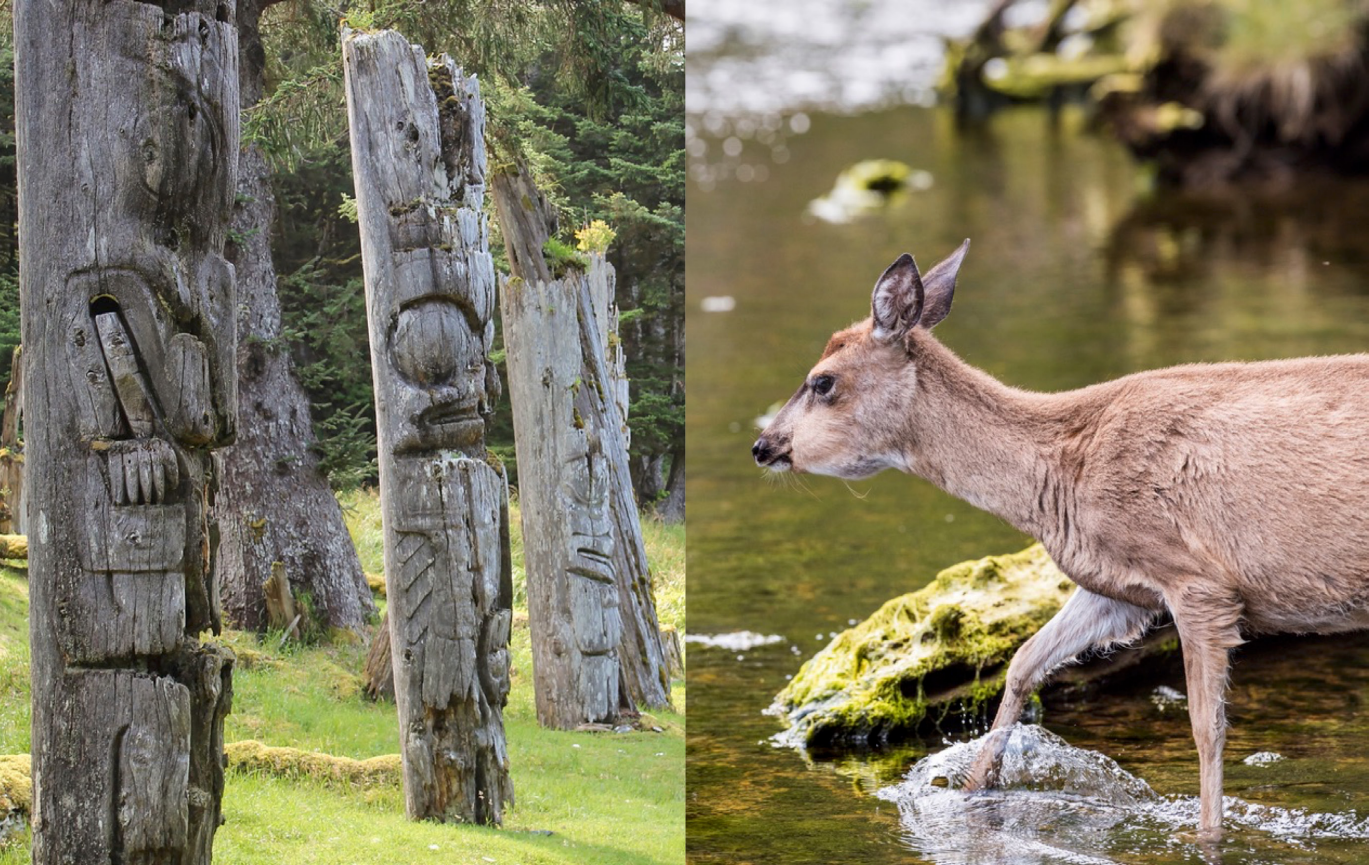
Totem carvings of the Haida people within Gwaii Haanas National Park Reserve and Haida Heritage Site on Haida Gwaii off the North Coast of British Columbia, Canada (left; photo credit: Parks Canada/Brady Yu). Over‐browse by invasive Sitka black‐tailed deer has caused severe ecological and cultural impacts throughout the archipelago, including threatening plant species that are highly valued within the Haida culture (right; photocredit: Parks Canada/Scott Munn)

In the Haida Gwaii archipelago (British Columbia, Canada), Sitka black‐tailed deer (*Odocoileus hemionus sitkensis*) is a widespread invasive species causing severe ecological and cultural impacts (Figure 1). Deer have disrupted ecosystem structure and dynamics due to their unconstrained browsing of native vegetation, preventing temperate rainforest regeneration; these impacts have been observed on numerous islands with deer present, which exhibit lower species richness compared to deer‐free islands across several taxa (Allombert et al., 2005; Allombert et al., 2005; Stockton et al., 2005). In addition, the Haida Nation have longstanding practical and spiritual relationships with several preferred food species of Sitka black‐tailed deer, such as western red cedar (*Thuya plicata*; Parks Canada and the Council of the Haida Nation, 2018). Given many islands with invasive deer lay within the boundary of Gwaii Haanas National Park Reserve, National Marine Conservation Area Reserve, and Haida Heritage Site (hereafter, Gwaii Haanas), Parks Canada and the Council of the Haida Nation have been working together to manage deer on ecologically and culturally significant islands within the park. Management of this highly mobile and invasive deer species could benefit from rapid and cost‐effective tools that provide individual‐ and population‐level information to guide levels of effort and approaches to reducing populations.

Genotyping‐in‐Thousands by sequencing (GT‐seq; Campbell et al., 2015) is a MTAS approach currently available that has seen widespread utility across studies in ecology, evolution, and conservation (Chang et al., 2021, 2022; Hayward et al., 2022; Jo et al., 2021; Natesh et al., 2019; Schmidt et al., 2020; Setzke et al., 2021; Sjodin et al., 2020). Here, we employed a scalable workflow to develop a GT‐seq panel for invasive Sitka black‐tailed deer in Gwaii Haanas. We used a modified protocol to successfully optimize the panel for use with noninvasive fecal and hair samples, demonstrating high levels of genotyping success and sample retention at hundreds of loci that were similar to those achieved with paired high‐quality deer tissue samples. We further provided a proof‐of‐concept application of our workflow to distinguish individuals, identify sex, and reconstruct kinship of Sitka black‐tailed deer from a World Heritage Site within Gwaii Haanas (SGang Gwaay), which is a focal island for ongoing deer management and ecological restoration (Parks Canada and the Council of the Haida Nation, 2018).

## 2 MATERIALS AND METHODS

### 2.1 Sample collection and DNA extractions

**FIGURE 2:**
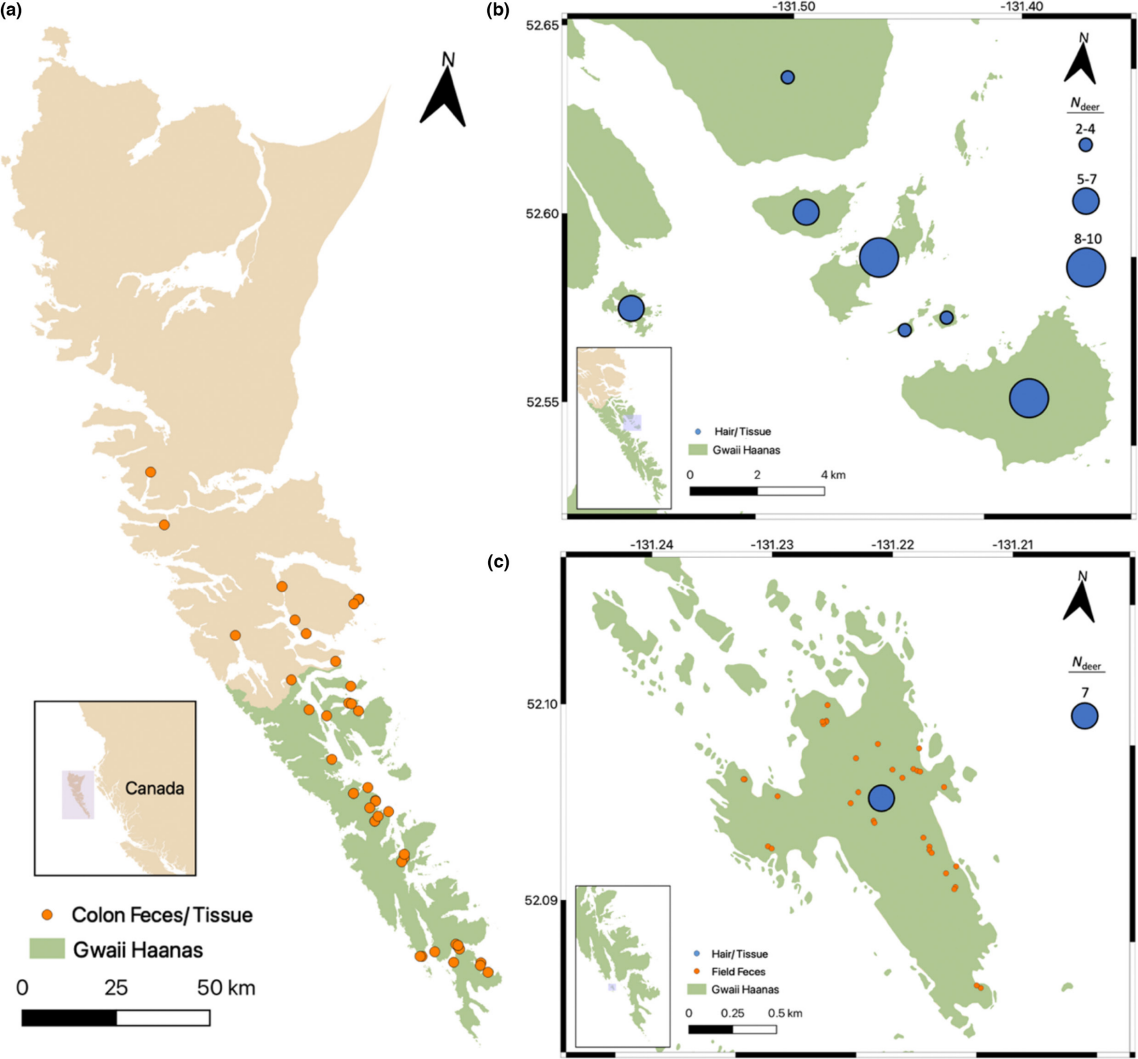
Sampling distribution of Sitka black‐tailed deer in Haida Gwaii, including Gwaii Haanas National Park (green). (a) Paired colon feces and ear tissue (orange; *n *= 46). (b) Paired hair and ear tissue (blue; *n *= 39). (c) The island of SGang Gwaay, where paired hair and ear tissue (blue; *n *= 7) were collected, as well as unpaired field feces (orange; *n *= 33). Locator maps are set in the bottom left of each panel

**FIGURE 3:**
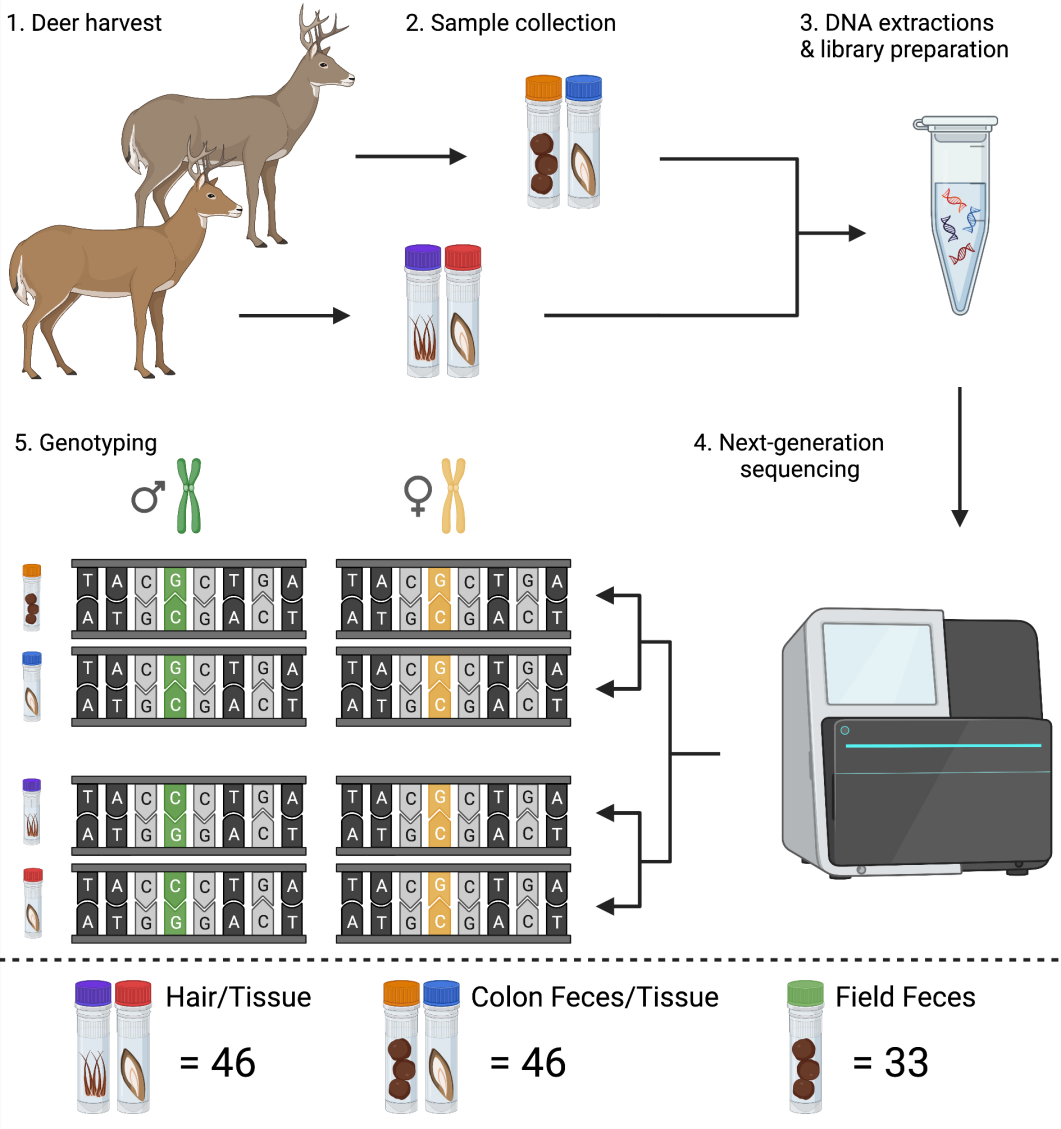
Paired sampling strategy demonstrating the transition from non‐invasive samples to genotypes using GT‐seq and the validation of non‐invasive samples with high‐quality tissue samples

Sitka black‐tailed deer were harvested by Parks Canada staff and contractors between 2017 and 2019 under a Parks Canada Agency Animal Care Permit from ecologically and culturally valuable islands in Haida Gwaii (Figure 2). Paired samples (colon feces/tissue, *n *= 46 each; hair/tissue, *n *= 46 each; Figure 3) from the same individuals were collected and stored dry (tissue/hair) or in 95% ethanol (tissue/feces) at −20°C. In 2020, a Parks Canada survey team collected fecal pellets (field feces, *n *= 33) on SGang Gwaay that were stored in 95% ethanol at 4°C. Sample collectors used alcohol wipes to sterilize hands and equipment, as well as pre‐filled tubes containing 95% ethanol. Hairs were pulled from deceased animals and tissue was cut from ears using sterile knives. Toothpicks were used for collecting deer fecal pellets from the ground. Feces were also collected immediately after death from the colons of deceased animals (i.e., colon feces), minimizing environmental exposure that can lead to DNA degradation.

Genomic DNA from ear tissue (10–20 mg) was extracted using the Qiagen DNeasy^®^ Blood and Tissue Kit following the manufacturer's protocol. Hair DNA extractions were performed using follicles (1.0–1.5 cm) from 10 to 15 hairs per individual and followed the user‐developed protocol available for Qiagen DNeasy^®^ Blood and Tissue Kits on the manufacturer's website. Fecal DNA extractions were performed using the outer shell of a single pellet per individual using the QIAamp^®^ DNA Fast Stool Mini Kit and manufacturer's protocol. All samples were treated with 4 μl of RNase A during extractions following manufacturer's protocol and quantified using a Qubit™ dsDNA High Sensitivity Assay Kit (ThermoFisher Scientific) and Qubit™ 3 Fluorometer (Life Technologies).

### 2.2 SNP selection for GT‐seq panel development

We used a previously generated restriction‐site associated DNA sequencing (RADseq; Baird et al., 2008) SNP dataset (*n *= 12,947; Burgess et al., 2022) to select candidate loci for GT‐seq panel development. Marker selection was optimized based on the informativeness of SNPs for: (1) individual identification; (2) pairwise kinship estimation; and (3) sex identification. We designed a sensitivity analysis to investigate the performance of SNPs binned in three minor allele frequency (MAF) categories (low = 0.00–0.10; moderate = 0.20–0.30; high = 0.40–0.49) to identify individuals and estimate pairwise kinship. In addition, we created a fourth category containing a proportional representation of loci from each MAF category that was observed in the original RADseq dataset. The number of loci per MAF category was varied by randomly selecting four subsets of SNPs: *n *= 50, 100, 200, and 400.

Each of the 16 SNP datasets was used to calculate the probability of identity (*P*_ID_) and probability of identity between siblings (*P*_IDSIB_) using GenAlEx v.6.5 (Peakall & Smouse, 2012). We compared the number of SNPs required from each MAF category to meet our designated threshold for acceptable individual identification where the probability of identification is less than the reciprocal of sample size (Peakall et al., 2006). Next, the ability of each dataset to estimate pairwise kinship was evaluated by employing the iRel r‐package (Gonçalves da Silva & Russello, 2011). Four replicates of each SNP dataset were used to simulate 1,000,000 dyads for each of two forms of first‐degree relatives (parent‐offspring, PO; full siblings, FS), second‐degree relatives (half siblings, HS), third‐degree relatives (first cousins, FC), and unrelated (UR). Relationship assignment accuracy was calculated and averaged using all dyads for each relatedness category.

To identify SNPs with sufficient power to identify sex, we used the RADseq dataset to calculate allele frequencies by locus separately for males (*n *= 90) and females (*n *= 75), implemented in VCFtools v.0.1.16 (Danecek et al., 2011). We selected loci with differences in allele frequency between sex (≥0.20) and validated their performance using the Bayesian clustering approach implemented in STRUCTURE v.2.3.4. (100,000 MCMC replicates, 100,000 burn‐in; Pritchard et al., 2000). For validation, we genotypically assigned 28 individuals of known phenotypic sex that were not among those used to generate the RADseq dataset.

DNA sequences containing candidate SNPs for GT‐seq panel development were sent to GTseek LLC (https://gtseek.com/) for subsequent primer design. Primers were designed to be 15–17 bases in length with a GC content between 20% and 80% and annealing temperature range between 58 and 63°C. The targeted amplicons were between 50 and 100 bases.

### 2.3 Panel optimization using high‐quality DNA samples

The initial GT‐seq library was constructed using high‐quality tissue samples from 92 individuals that were previously genotyped using RADseq (Burgess et al., 2022). These individuals all had paired noninvasive samples (*n*_hair _= 46, *n*_colon feces _= 46), represented a ~1:1 male/female sex ratio, and maximized the geographic representation of the dataset (Figure 2). We included four replicates that were previously used to estimate RADseq genotyping error (Burgess et al., 2022), enabling comparison between the two genotyping methods.

Library construction followed the original protocol described in Campbell et al. (2015) with a few modifications. Prior to PCR1, all DNA samples were standardized at 15 ng/μl to help obtain an equal distribution of amplicons across samples. Each PCR1 reaction contained 3.5 μl of Qiagen^®^ Multiplex PCR Master Mix, 1.5 μl of pooled primers, and 2 μl of DNA template before thermocycling [95°C–15 min; 5 cycles (95°C–30 s, 5% ramp down to 57°C–30 s, 72°C–2 min); 10 cycles (95°C–30 s, 65°C–30 s, 72°C–30 s); 4°C hold]. The PCR1 products were diluted 1:20 and 3 μl was used from each sample for amplification in PCR2. Next, PCR2 products were quantified with Quant‐iT™ PicoGreen™ dsDNA assays (Invitrogen) using a Viia7 real‐time PCR system (Life Technologies). PCR products were normalized to 20 ng/μl before pooling 5 μl from each sample for library purification using the MinElute^®^ PCR Purification Kit. Purified libraries (*n *= 4; see below) were eluted into 24 μl of nuclease‐free water and sequenced using one Mid Output Reagent Kit (300 cycles) per library on an Illumina MiniSeq™.

Using the GT‐seq bioinformatics pipeline (https://github.com/GTseq), raw sequencing reads were demultiplexed into individual.fastq files and genotyped (*GTseq_Genotyper_v3*.*pl*). Panel optimization was largely guided by the outputs of *GTseq_SeqTest*.*pl* and *GTseq_Primer*‐*Interaction*‐*Test*.*pl*; the former identified overrepresented primers based on the proportion of total reads that contained a forward primer, while the latter identified primers that were responsible for copious primer‐dimers or undesirable PCR artifacts. All primers found to be negatively affecting genotyping success were omitted from a second GT‐seq library, which was constructed, sequenced, and genotyped following the same procedure described above for the initial library, including identical high‐quality DNA samples.

### 2.4 Panel optimization using noninvasive DNA samples

A third GT‐seq library was prepared using noninvasive samples (*n*_hair _= 46, *n*_colon feces _= 46) that were paired with previously genotyped high‐quality tissue samples and 33 field fecal samples collected from SGang Gwaay (Figure 3). The library also included four replicates of each sample type (e.g., hair, colon feces, field feces) for a total of 137 samples. Library construction was identical to previous libraries except samples were standardized at 3 ng/μl when possible due to low DNA concentrations in noninvasive samples after extraction. To account for the degraded DNA template of low concentrations, we performed PCRs twice using separate i7 primers and combined the resulting sequence data bioinformatically.

Given poor genotyping of the third library (see Results), we constructed a fourth library using the same set of noninvasive samples, including four replicates of each sample type. To further account for low DNA concentrations in the noninvasive samples, we made additional modifications to the original GT‐seq protocol. First, we divided our primers into two separate pools to reduce the number of primers in each multiplex PCR1. We then made revisions to the PCR1 protocol; each reaction included 5 μl of Qiagen^®^ Multiplex PCR Master Mix, 1 μl of primer pool, and 4 μl of DNA template, which were subjected to the following thermocycling conditions: 95°C–15 min; 35 cycles (94°C–30 s, 60°C–90 s, 72°C–60 s); 60°C–30 min; 4°C hold. The final library was sequenced and then genotyped using the GT‐seq bioinformatics pipeline as detailed above.

### 2.5 Panel validation for invasive species management applications

After the initial genotyping of each library, samples were removed if they yielded >50% missing data. The percentage of genotyping success was then calculated by locus and those with >50% missing data were excluded from the final SNP dataset. Genotyping error was measured as the discordance of genotypes between replicate samples and was calculated both within‐sample types and across‐sample types using paired samples. Genotyping success and error were also calculated across sequencing methods by comparing the final GT‐seq SNP dataset to the same sites generated using RADseq.

To validate our final GT‐seq panel for the targeted invasive species management applications of individual identification, pairwise kinship estimation, and sex identification, we performed comparative analyses between the noninvasive samples genotyped using GT‐seq and their high‐quality pairs originally genotyped using RADseq. GenAlEx was used to calculate *P*_ID_ and *P*_IDSIB_ and determine the number of unique individuals. Pairwise kinship was calculated between individuals in GenoDive v.3.0 (Meirmans & Van Tienderen, 2004) using the relative probability of allelic identity‐by‐descent (Loiselle et al., 1995). Finally, we employed Bayesian clustering (Pritchard et al., 2000) of the retained sex‐associated SNPs to assign sex of noninvasive samples collected from individuals of known sex in the RADseq dataset. As a proof‐of‐concept application, the same analyses were used to identify and sex individuals and estimate pairwise kinship using the field fecal samples from SGang Gwaay (*n *= 33).

## 3 RESULTS

### 3.1 SNP selection

**FIGURE 4:**
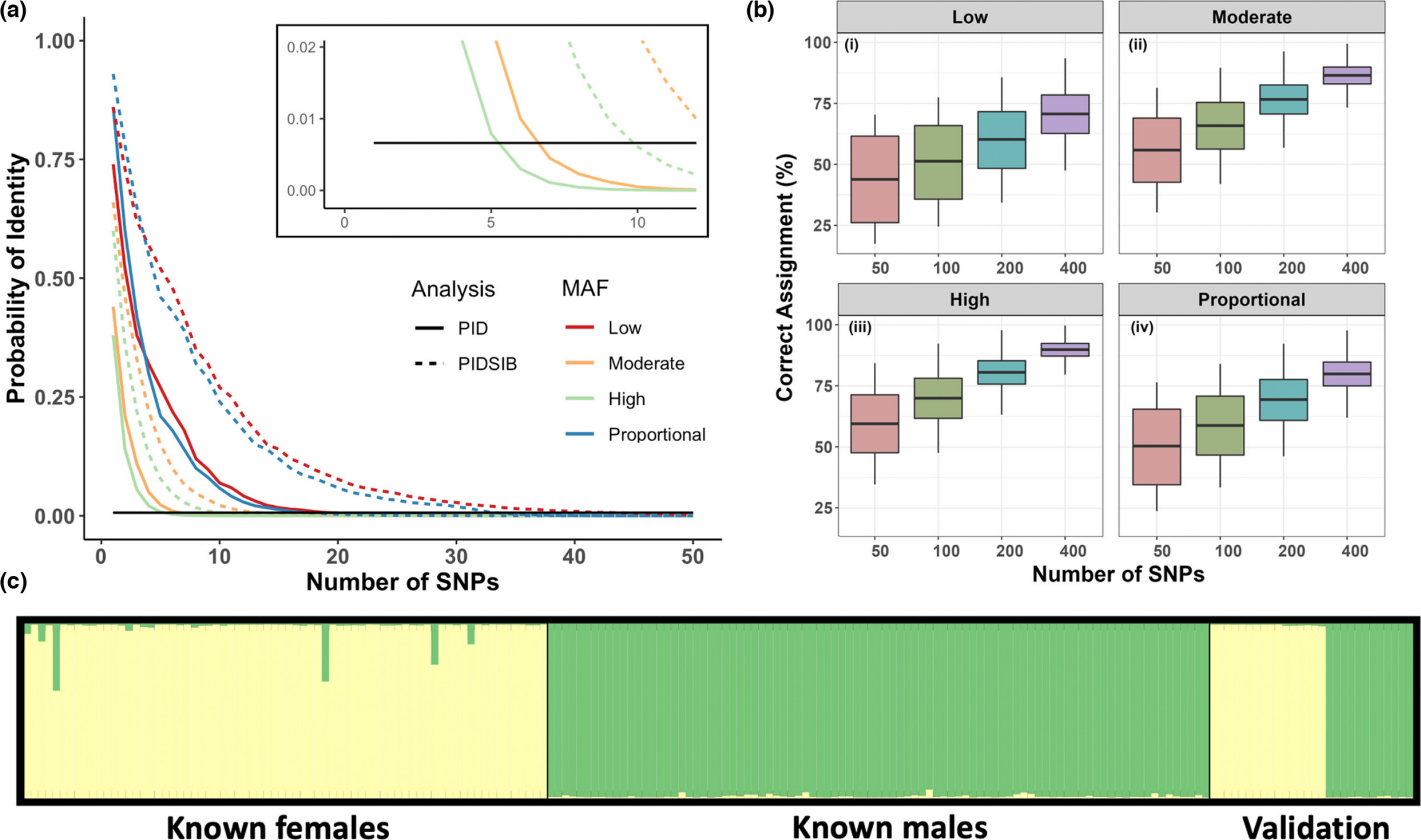
Analyses to inform GT‐seq SNP selection for Sitka black‐tailed deer. (a) Probability of identity (*P*_ID_) and probability of identity of siblings (*P*_IDSIB_) calculated using loci of varying minor allele frequency (MAF). The horizontal black line represents the threshold of acceptable individual identification (0.007). (b) Mean probability of correctly identifying first‐degree (full‐sibling), second‐degree (half‐sibling), third‐degree (first cousin), and unrelated relationships using varying numbers of loci across different MAFs. (c) Sex assignment using Bayesian clustering of eight sex‐associated loci

Across both individual identification and pairwise kinship analyses, high MAF SNPs consistently performed better than the other categories. For individual identification, high MAF SNPs were most efficient at calculating *P*_ID_ and *P*_IDSIB_, requiring only 6 and 10 SNPs, respectively; however, our results indicated that 40 SNPs of any MAF were able to meet this threshold (Figure 4a). For the pairwise kinship estimations, high MAF SNPs exhibited the greatest assignment accuracy, which improved with increasing numbers of SNPs across all categories (Figure 4b). Eight SNPs were identified as sex‐associated candidates and successfully assigned sex in all 193 deer of known phenotypic sex (Figure 4c).

Based on these results, we submitted DNA sequences for GT‐seq primer design for 466 high MAF SNPs, plus eight sex‐associated SNPs. Of these 474 candidate SNPs, primers were successfully designed for 369 after in silico testing, including for all eight sex‐associated SNPs.

### 3.2 Panel optimization

Sequencing of our first GT‐seq library resulted in 10,925,017 paired‐end reads, but genotyping percentage across all loci was low (12.5%). We removed 39 loci that were negatively impacting genotyping success, leaving 330 for construction of our second library. Sequencing of this library resulted in 12,563,048 paired‐end reads and genotyping percentage across all loci improved to 86.5%. A single sample was removed due to >50% missing data. An additional 22 loci were removed during optimization (i.e., the corresponding primers were over‐amplifying and taking up a disproportionate number of reads), resulting in a panel of 308 SNPs that included 7 sex‐associated SNPs.

**FIGURE 5:**
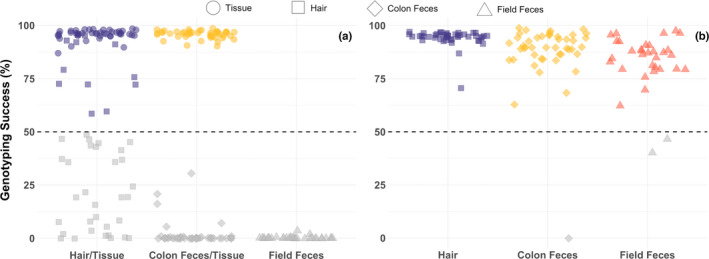
GT‐seq genotyping success at 244 loci across individuals grouped by sample type, which are represented by distinct shapes within inset legend. (a) Samples genotyped using the original GT‐seq protocol, including paired hair and tissue (*n *= 46 each), paired colon feces and tissue (*n *= 46 each), and field feces (*n *= 33). (b) Identical non‐invasive samples as in (a) but instead genotyped using our revised PCR1 conditions, including hair (*n *= 46), colon feces (*n *= 46), and field feces (*n *= 33). The dotted lines represent our threshold for acceptable missing data (50%); samples removed due to high missing data are colored grey

The third library that was comprised of noninvasive samples yielded 17,155,628 paired‐end reads; however, the mean percentage of on‐target reads across samples was only 1.52%. Few samples (10.4%) genotyped at >50% of loci, including 12/46 (26.1%) hair samples, 1/46 (2.2%) colon fecal sample, and none of the field fecal samples (Figure 5a). After applying our revised PCR1 conditions to the same sample set, sequencing of the fourth library yielded 10,719,923 paired‐end reads with 51.8% being on target. All hair samples genotyped at >50% of loci and only three fecal samples (one colon and two field) failed to genotype at >50% of loci, resulting in 122 of 125 samples retained (97.6%) for downstream analysis. Sixty‐four loci were removed due to >50% missing data, resulting in a final SNP dataset of 244 loci.

### 3.3 Panel validation and application

Overall average genotyping success of retained noninvasive samples was high (90.3%) and comparable to that of high‐quality tissue samples genotyped using both GT‐seq (95.7%) and RADseq (92.4%; Table 1; Figure 5b). Within noninvasive sample types, genotyping success was 94.1% for hair, 89.4% for colon feces, and 85.6% for field feces. Mean sequencing depth was 176 for hair, 119 for colon feces, and 96 for field feces. Genotyping error was much lower (<0.8%) within GT‐seq samples, regardless of sample type, than in high‐quality RADseq samples (6.0%; Burgess et al., 2022). Genotyping discordance between 45 paired tissue and hair samples was 0.27%, while genotyping discordance between 43 paired tissue and fecal samples was 0.74%. Bayesian clustering using the seven retained sex‐associated SNPs accurately determined the sex of all test samples of known phenotypic sex (100%).

**TABLE 1:** A comparison of genotyping at 244 SNP loci across methods and sample types for Sitka black‐tailed deer

Method	Sample type	*n*	% of retained *n*	Mean depth	Genotyping success %	Genotyping error %
RADseq	Tissue	92	100	23 ± 16	92.4	6.02
GT‐seq	Tissue	92	98.9	149 ± 63	95.7	0.11
	Hair	46	100	177 ± 35	94.1	0.33
	Feces (all)	79	96.2	110 ± 40	87.9	0.39
	Colon	46	97.8	119 ± 39	89.4	0.78
	Field	33	93.9	96 ± 37	85.6	0.00
	Total	217	98.2	141 ± 56	92.6	0.31

We identified 20 unique genotypes from the 33 field fecal samples collected on SGang Gwaay. In some cases, different pellets belonging to the same individual were geographically located within 5 m of each other, while others were found on opposite sides of the island (~650 m apart) demonstrating successful genetic mark‐recapture. All identified individuals from SGang Gwaay exhibited high levels of pairwise kinship (mean = 0.20). Thirty‐two of 33 field‐collected feces were sexed, as one sample was missing data at all sexing loci; of the 20 identified individuals, 9 were females and 11 were males.

## 4 DISCUSSION

Multiplexed targeted amplicon sequencing approaches, such as GT‐seq, have begun to overcome challenges related to data quality and quantity that are characteristic of noninvasive samples, allowing investigators to harness the power of massively parallel DNA sequencing for addressing relevant questions in ecology, evolution, and conservation (Meek & Larson, 2019; Natesh et al., 2019; Schmidt, Campbell, et al., 2020). Yet, there are still inefficiencies when applying GT‐seq to noninvasive samples that can lead to sample loss, marker dropout, and degraded quality that can limit the informativeness of resulting genotypic data and the associated robustness of downstream analyses. Here, we optimized a GT‐seq panel for utility with noninvasive fecal and hair samples; by adjusting the PCR1 conditions to account for low‐quantity and degraded DNA, we demonstrated high sample retention (97.6%), genotyping success (90.3%), and genotyping concordance (99.5%) relative to those produced from high‐quality tissue sample at the same panel of 244 SNPs. Importantly, the adjustments we made did not require additional laboratory equipment or expertise, and maintained the rapidity and cost‐effectiveness that has elevated GT‐seq as an MTAS approach of choice in molecular ecology (Eriksson et al., 2020; Hayward et al., 2022; Schmidt, Campbell, et al., 2020).

Another advantage of GT‐seq is the ability to design panels that can simultaneously provide insights at multiple scales by strategically targeting SNPs of varying signal. To maximize the effectiveness of multi‐scaled panels, it is important to conduct sensitivity analyses in order to determine the number and characteristics of component SNPs, and perform validation to ensure the desired applications are feasible. We designed our SNP selection and validation workflow to construct a GT‐seq panel for effectively identifying and sexing individuals, as well as estimating pairwise kinship up to third‐order relationships. Other recent applications of multi‐scaled GT‐seq panels include identifying individuals and reconstructing population structure in polar bears (Hayward et al., 2022) and rattlesnakes (Schmidt, Govindarajulu, et al., 2020), and identifying life history forms, assigning stock, and reconstructing population history in kokanee salmon (Chang et al., 2022; Setzke et al., 2021).

Noninvasive genetic sampling has been frequently used to monitor at‐risk or elusive species for conservation, yet also has the potential to directly inform the management of invasive species. eDNA has been demonstrated as an effective noninvasive approach for invasive species research; however, such approaches are largely limited to presence‐absence detection (Beng & Corlett, 2020). Often, invasive species management can benefit from population‐level information, especially when planning eradications or culls (Browett et al., 2020; Burgess et al., 2021). For example, estimating population densities before a cull can help to inform stopping rules when target densities are reached, preventing excess resource investment (Ramsey et al., 2011). Alternatively, cataloging the number of remaining individuals during eradications can help decide when an eradication has been successfully completed (Macdonald et al., 2019).

As a proof‐of‐concept, we applied our GT‐seq panel to field‐collected fecal samples of invasive Sitka black‐tailed deer from a World Heritage Site of high management priority (SGang Gwaay), where deer have long been targeted for management, with population culls occurring between 1998 and 2003 and again in 2018. Our panel successfully demonstrated individual identification, genetic mark‐recapture, sexing, and kinship estimation, and will be used to inform future management operations of deer on SGang Gwaay by providing individual and population information before, during, and after culls or eradications. For example, prior to a planned eradication, management can perform fecal surveys across the island to estimate the number of unique individuals present, helping to gauge effort requirements. A major obstacle to successful eradication is the removal of deer at low densities, as individuals can become elusive and have even been observed changing their behavior in response to hunting, leading to significantly higher costs of removal per individual (Macdonald et al., 2019). By genotyping deer harvested during the later stages of an eradication, hunters can be informed of remaining numbers and change tactics accordingly. Furthermore, if an eradication fails, genotyping deer on SGang Gwaay following the eradication could provide insights into the cause(s) of failure, whether due to survivors or re‐invaders from neighboring Moresby Island. Beyond the demonstrated application to deer on SGang Gwaay, our GT‐seq panel was designed to be informative across Gwaii Haanas, which provides flexibility if additional islands emerge as new targets for management. Previous population genetic and genomic studies showed that deer in Gwaii Haanas were frequently dispersing between proximal islands (Burgess et al., 2022; Reimchen et al., 2008); future use of this rapid and cost‐effective GT‐seq panel will allow invasive species managers to design efficient biosecurity responses and enact timely decisions toward control or eradication operations.

## AUTHOR CONTRIBUTIONS

**Brock T. Burgess:** Conceptualization (supporting); Data curation (lead); Formal analysis (lead); Investigation (lead); Writing – original draft (lead); Writing – review & editing (equal). **Robyn L. Irvine:** Conceptualization (supporting); Funding acquisition (supporting); Resources (supporting); Supervision (supporting); Writing – review & editing (supporting). **Michael A. Russello:** Conceptualization (lead); Funding acquisition (lead); Investigation (supporting); Project administration (lead); Resources (lead); Supervision (lead); Writing – original draft (supporting); Writing – review & editing (equal).

## CONFLICT OF INTEREST

None declared.

## Data Availability

Illumina raw reads are available from the NCBI sequence read archive (BioProject ID: PRJNA831890); SNP genotypic data are deposited in the Dryad Digital Repository (doi.org/10.5061/dryad.vq83bk3vs).
